# Efficient gene delivery and silencing of mouse and human pancreatic islets

**DOI:** 10.1186/1472-6750-10-28

**Published:** 2010-03-30

**Authors:** Bruno Lefebvre, Brigitte Vandewalle, Justine Longue, Ericka Moerman, Bruno Lukowiak, Valery Gmyr, Kathrin Maedler, Julie Kerr-conte, François Pattou

**Affiliations:** 1 Univ Lille Nord de France, F-59000 Lille, France; 2 INSERM U859, F-59000 Lille, France; 3 CHU Lille, F-59000 Lille, France; 4 Center for Biomolecular Interactions Bremen, University of Bremen, Germany

## Abstract

**Background:**

In view of the importance of beta cells in glucose homeostasis and the profound repercussions of beta cell pathology on human health, the acquisition of tools to study pancreatic islet function is essential for the design of alternative novel therapies for diabetes. One promising approach toward this goal involves the modification of gene expression profile of beta cells.

**Results:**

This study describes a new method of gene and siRNA delivery into human pancreatic islets by microporation technology. We demonstrated that mild islet distention with accutase greatly enhanced the transfection efficiency without compromising in vitro function (secretion, apoptosis and viability). As an example, the recently identified gene involved in type 2 diabetes, ZnT8, can be over-expressed or silenced by RNA interference using this technology. Microporation can also be used on rodent islets.

**Conclusions:**

Taken together, our results demonstrate that microporation technology can be used to modify gene expression in whole rodent and human islets without altering their in vitro function and will be key to the elucidation of the factors responsible for proper islet function.

## Background

Establishing robust gene delivery systems that result in efficient transfection of the pancreatic-beta cells is a prerequisite to the study of type 1 and 2 diabetes and may also represent a critical step toward clinical application. Numerous report in the literature describe the application of gene over-expression and silencing in beta-cells derived cell-lines to study beta-cell function and the mechanisms behind type 1 and 2 diabetes pathologies. However, the findings in beta cell lines are not always congruous with results in primary islet beta cells, underlying the necessity of studying beta-cells in their native environment [1,2].

Modulation of gene expression in the context of intact pancreatic islets is particularly difficult because of their three dimensional structure (50-500 μm in diameter), making physical access to the islet core difficult. Islets are organized in organoid cell clusters of 1000 to 2000 cells made up of four types of endocrine secreting cells, the insulin containing β-cells, the glucagon containing α-cells, the somatostatin containing δ-cells and the pancreatic polypeptide-producing (PP) cells [3]. Over the past decades, numerous attempts have been made to transfect or transduce intact cells using lipid-mediated plasmid delivery [4,5], adenovirus vectors [6-8], lentivirus vectors [9] and magnetic nanocarriers [10]. However, the major obstacle of these studies is that only cells at the periphery of islets become efficiently transfected or transduced.

In the present study, we herein demonstrate successful transfection of plasmids and siRNA to whole suspension cultured rodent and human pancreatic islets. First, a mild digestion with accutase enzyme was found to be necessary to predistend (ie without dissociation) human islets and enhance accessibility to core cells. Subsequently, rodent islets or predistended human islets were subjected to microporation in the presence of the plasmid of interest or siRNA. Using GFP vector and confocal microscopy, we have been able to show that most of the rodent and human islet cells (>70%) have been efficiently transfected. Furthermore, we demonstrated that the viability and insulin secretory function of the human transfected islets were not altered in that they responded normally to a glucose challenging. As an example, efficacy of transfection and silencing in human islets has been confirmed using the recently identified diabetes-linked zinc transporter ZnT8. Taken together, our results demonstrated that microporation of rodent islets or human islets after predistension is a new robust tool to study gene functions in the context of whole islets.

## Results

### Demonstration of human core islet targeting after accutase treatment

**Figure 1:**
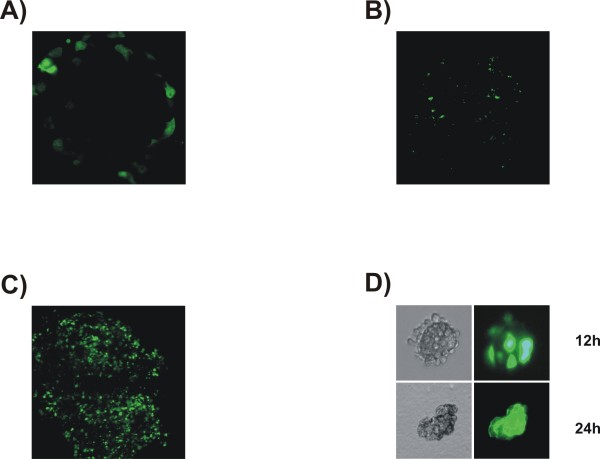
**eGFP expression in human and mouse islets following adenoviral and microporation transfection delivery**. (A) GFP expression of human islets infected with Ad-GFP. (B) GFP expression of human islets microporated with an eGFP plasmid. (C) GFP expression of human islets pretreated with accutase and microporated with an eGFP plasmid. (D) GFP expression of mouse islets microporated with an eGFP plasmid by fluorescent microscopy 12 or 24 h after microporation.

To evaluate the transfection efficiency, confocal microscopy and estimation of islet viability and metabolism were used. Adenoviruses (Ad)-GFP or transfections were carried out as described in Materials and Methods. As shown in Figure 1A, adenovirus was able to infect the intact human islets but eGFP staining (2 day post-transduction) revealed only surface expression, as previously reported [11]. In addition, we found a significant decrease in the ATP level suggesting that adenoviral transduction altered islet viability (data not shown).

Electroporation (EP) or microporation (MP) technology demonstrated to be highly efficient in primary cells and cell lines that are very difficult to transfect [12]. As shown in Figure 1B, MP of intact islet with eGFP plasmid led to very low transfection efficiency (2 days post-transfection). The same results were found for different electrical parameters (data not shown). However, pretreatment with accutase greatly enhanced the number of GFP positive cells with a staining of peripheral cells as well as core islets (Figure 1C). Using the same parameters, 70% of mouse islet cells were found to be transfected observed by eGFP staining without predistention step (Figure 1D).

### Viability and human islet function were not impaired after accutase pretreatment and transfection

**Figure 2:**
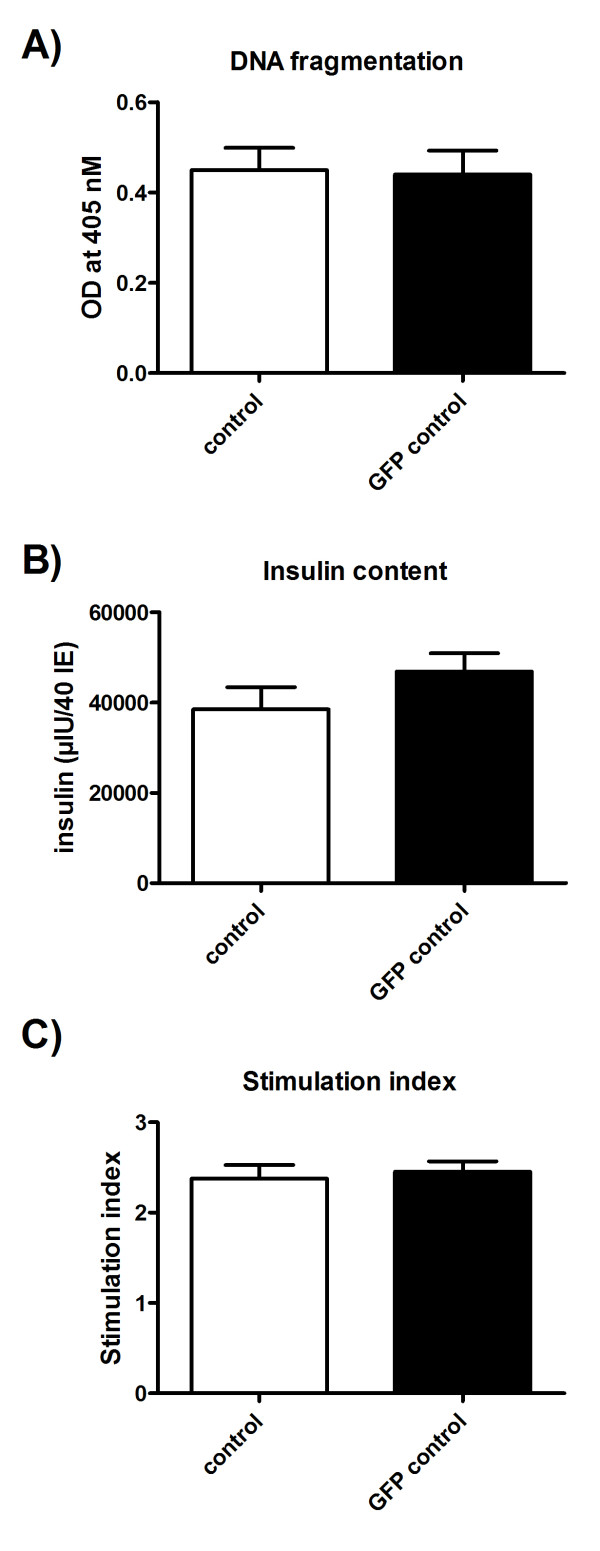
**Human islet metabolic parameters were not modified upon accutase pretreatment and microporation**. (A) apoptotic index, (B) insulin content and (C) stimulation index were measured in control human islets or overexpressing GFP-human islets after 48 h culture. Values are means +/- SEM of islets from n = 3 pancreases.

To determine whether gene transfer using accutase pretreatment and microporation altered islet viability, we first used trypan blue staining, ATP level and DNA fragmentation as indicators of viable cells. No significant differences were found in the viability, ATP level (data not shown) and apoptotic index (Figure 2A) between control islets and 2 day post-transfected islets after accutase treatment.

Islets regulate insulin release physiologically in response to glucose. To determine if MP of islets after accutase pretreatment maintains normal regulated insulin secretion, an essential requirement for any gene function studies, insulin content and glucose stimulated insulin secretion were determined on control and 2 day post-transfected islets. As shown in Figure 2B and 2C, both insulin content and stimulation index were comparable between the two conditions.

These results demonstrated that accutase pretreatment combined with MP of islets does not affect the viability and function of human islets.

### Validation of gene transfer and silencing efficacy by Quantitative RT-PCR

**Figure 3:**
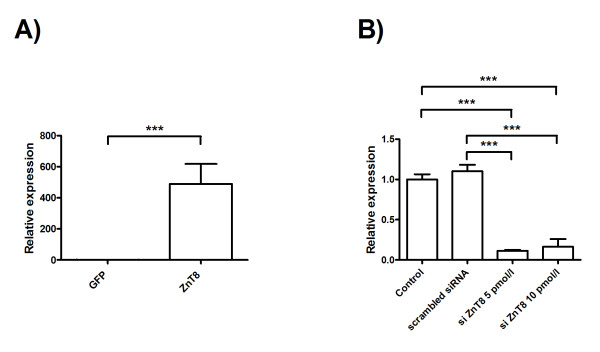
**Real time PCR analysis of ZnT8 expression in (A) control islets or overexpressing ZnT8 islets and (B) control islets or microporated with 5 pM or 10 pM of siZNt8**. Basal ZnT8 expression was arbitrarily set to 1. Values are means +/- SEM of islets from n = 3 pancreases.

Final validation of gene transfection and silencing efficacies was obtained by quantitative RT-PCR for zinc transporter ZnT8. Level of ZnT8 expression was first assessed on transfected human islets with eGFP or eGFP-ZnT8 plasmid. After two days, we observed a 500-fold increase in the level of ZnT8 mRNA as compared to control (Figure 3A). A significant > 70% reduction in endogenous ZnT8 mRNA transcript levels was observed in human islets transfected with the siZnT8 (5 and 10 pmol/l) as compared with control islets or transfected with scrambled siRNA after 4 days.

Taken together, these data demonstrate that accutase pretreatment and MP is an efficient method for gene expression and silencing in human islets.

## Discussion

As the beta-cell plays a central role in both type 1 and type 2 diabetes, the acquisition of tools to study beta cell function represents a key research for developing alternative therapies. One approach used was the development of beta-cells derived cell-lines. Although they represent valuable tools for the study of molecular events underlying beta-cell function and dysfunction, the behavior of these cell lines does not perfectly mimic the beta-cell physiology [1]. To date, most islet transfections have been performed using viral vectors. However, limited loading capacity of genes has been observed in most of the cases [13,14]. In addition, our results suggested that adenovirus transduction perturbed human islet viability. Liposomal gene carriers have often resulted in ineffective gene expression, islet toxicity at high liposome/plasmid ratios and poor insulin secretion after transfection [5]. The disadvantages of viral or liposomal vectors have triggered us to the development of alternative methods. First, to overcome the limited loading capacity of genes in human islets, we used mild digestion with accutase. By using this pretreatment, we achieved much higher transfection efficiency for all non-viral transfection procedure tested. Efficient delivery in mouse islets did not require predistention and may be related to species differences in regard to cytoarchitecture [3]. In addition, we demonstrated that islet viability and function were not affected by this procedure. This distension technique was previously described in our lab to improve penetration of Newport Green, a fluorescent zinc chelator into the human islet core [15]. Second, to overcome islet toxicity often observed during the transfection procedure, we used an electroroporation-based technology. We choose to test microporation technology, a capillary tip and pipette-based gene transfer technique which lacks the deleterious effects of cuvette-based electroporation methods including pH variation, temperature increase, turbulence, and generation of metal ions as described by NanoEnTek Inc (Seoul, Korea). In addition, it has been shown that this technology has beneficial features for transfecting slowly dividing cells such as primary cells [12]. By using the combine methodology of accutase pretreatment and microporation, we have been able to achieve high transfection efficiency without affecting human islet survival and function.

## Conclusion

We found that transfection of human islets using accutase distention and microroporation can achieve efficient gene expression and silencing without impairing islet function and represents a suitable strategy to study gene function in human islets. Microporation also works in rodent islets.

## Methods

### Human and mouse islet isolation

Human pancreases were harvested from adult brain-dead donors in accordance with French Regulations and with the local Institutional Ethical Committee. Clinical grade pancreatic islets in number insufficient for transplantation were isolated as described [16]. Islet number was determined on samples of each preparation after dithizone staining and expressed as equivalent number of islets (IE) [17]. All experiments were realized at least on 3 different donors of >80% purity. A total of 9 human islet preparations were used for this study.

All animals were housed in a temperature-controlled room with a 12-hour light/dark cycle and were allowed free access to food and water conformed to guidelines of the Bremen Senate for Work, Health, Women, Youth and Social Affairs and from the Bremen Senate in agreement with the National Institutes of Health animal care guidelines and §8 of the German animal protection law. Mouse islets from C57Bl/6J mice were isolated and cultured as described previously [18]. The islets were then pre-cultured in RPMI-1640 medium containing 11.1 mM glucose, 100 U/ml penicillin, 100 μg/ml streptomycin and 10% FCS (Invitrogen, Scotland)

### Cell culture and transfection

Purified human islets were cultured in CMRL 1066 medium (Gibco BRL, Life Technologies) containing 0.6% BSA (Roche Diagnostics, France), penicillin (100 μUI/ml), streptomycin (100 μg/ml) [19].

Human islet transfections were performed using microporation (Microporator MP100, Digital Bio, Labtech France) after accutase (PPA Laboratories GmbH, Austria) pretreatment (2500 IE incubated at 37°C for 2 min). Transfection experiments were performed between 18 h to 3 day post-isolation.

In a preliminary study, we tested in terms of islet function (insulin secretion), apoptosis and viability the 24 different experimental microporation protocols proposed by the manufacturer Digital Bio (South Korea) using an eGFP plasmid and determined the optimal program: 2 pulses of 1400 V, 20 ms with 1 μg of plasmid for 2500 IE for human islets.

For ZnT8 studies, control eGFP or ZnT8-eGFP (kind gift of Dr Chimienti) or siRNA to ZnT8 obtained from Dharmacon (Perbio Science, France) were microporated according to the above protocol.

As a comparison adenoviral transduction (adenoviral (Ad)-GFP) was performed as follows:2500 IE placed in the CMRL BSA medium, were transduced (MOI 60) for 4 hours. The islets were then washed with medium and incubated for an additional 48 h.

For mouse islets, islets were transferred into a 24-well plate 24 h after isolation and transfected with 2 μg/ml CMV-eGFP using a standard program of 30 ms, 2 bursts and 950 V with Microporator MP100 according to the manufacturer's instructions. Transfection efficiency was monitored at 12-24 h by analyzing GFP-positive islet cells under a fluorescent microscope Nikon MEA53200 (Nikon GmbH Duesseldorf, Germany) and images were acquired using NIS-Elements software (Nikon).

### Estimation of islet cell viability

Islet viability was assessed after dithizone and trypan blue staining on 3 aliquots of 80 IE per condition.

ATP content was measured on 3 aliquots of 40 IE per condition by a luminescence ATP detection assay system as described by the manufacturer (ATPlite, Perkin Elmer).

### Insulin content and glucose stimulated insulin release (GSIS)

Insulin content was assayed using a radioimmunoassay kit (CIS bio International) in the lysate of 3 aliquots of 40 IE per condition. GSIS was determined by static incubations of islets for 1 hour with low glucose (2.8 mmol/l, basal) followed by 1 hour with high glucose (20 mmol/l, stimulated). Stimulation indexes defined as the ratio of stimulated to basal insulin release were estimated on 5 aliquots of 40 IE per condition.

### Determination of DNA fragmentation

The specific determination of mono-and oligonucleosomes in the cytoplasmic fraction of islet lysates (3 aliquots of 160 IE per condition) was achieved by quantitative sandwich enzyme-immunoassay using mouse monoclonal antibodies against DNA and histones (cell death detection ELISA kit from Roche Molecular Biochemicals).

### RNA preparation and real-time PCR

Total RNA was prepared using RNeasy Minikit (Qiagen). Purified RNA was adjusted to 1 μg/μl and its integrity was assessed with the Agilent RNA 6000 chips coupled with the Agilent 2100 Bioanalyzer (Agilent Technologies), by visualizing the 18S and 28S ribosomal ribonucleic acid (rRNA). Reverse transcription (RT) was performed using random hexamers as recommended by the manufacturer (Applied Biosystems). cDNAs were analyzed by PCR amplification using the TaqMan PCR master mix (Applied Biosystems) and a mix of RPLO primers and probes. The different probes were purchased from Applied Biosystems (assay on demand kit). Reactions (40 cycles) and data analysis were carried out with an ABI Prism 7900 (Perkin-Elmer).

### Statistical study

Results are presented as means ± SEM. The statistical differences between the groups are analyzed with one-way ANOVA and the Fisher's Least Significant Difference test using Statview 4.1 software (Abacus, Berkeley, CA).

## Authors' contributions

BL designed research, performed research and wrote the paper. KM designed research and performed research. BV, JKC and FP designed research. JL, BL, EM, VG performed research.

All the authors have read and approved the final manuscript.
